# A new hypothesis on HIV cure

**DOI:** 10.12688/f1000research.4529.1

**Published:** 2015-03-24

**Authors:** Florian Hladik

**Affiliations:** 1 Vaccine and Infectious Disease Division, Fred Hutchinson Cancer Research Center, Seattle, Washington, 98109, USA; 2 Departments of Obstetrics and Gynecology, and Medicine, University of Washington, Seattle, Washington, 98195, USA

**Keywords:** AIDS, HIV Latency, NRTIs, Tenofovir, Antiretroviral drugs, Mucosa, Berlin patient, Integrase Inhibitors

## Abstract

In this opinion article, I provide the rationale for my hypothesis that nucleoside reverse transcriptase inhibitors (NRTIs) may prevent human immunodeficiency virus (HIV) cure by promoting the survival of cells with integrated provirus. If correct, we may be closer to a cure than we realize.

## Introduction

Current antiretroviral treatment (ART) is extremely effective in controlling replication of human immunodeficiency virus (HIV) and in many patients suppresses the number of virions measurable in peripheral blood, i.e., the HIV viral load, to undetectable levels. Nevertheless, whenever ART is stopped, HIV levels rebound and the disease returns. This lack of eradication is attributed to a stable latent reservoir of HIV-1 in resting CD4 ^+^ T lymphocytes and perhaps other susceptible cell types such as macrophages ^1^. These cells harbor HIV in the form of replication-competent proviruses that are integrated into the host genome. During effective ART this reservoir decays so slowly that it would theoretically require treatment for 60 years or longer to eliminate it ^2^.

For this reason, HIV-related research efforts are increasingly being devoted to understanding the nature of this latent virus reservoir and how to eradicate it. Two aspects of the latent virus reservoir have emerged as crucial in maintaining infection. First, HIV is not transcribed or translated from latently infected cells, allowing them to escape detection from the immune system. Second, cells with integrated provirus persist and even expand despite continuous ART ^3– 5^. To circumvent viral persistence, “kick and kill” strategies have been proposed that attempt to reactivate HIV with latency-reversing agents and then destroy these cells with the help of targeted active or passive immunization strategies. Unfortunately, reactivating cells from infected individuals *ex vivo* has thus far not shown promising results ^6, 7^ and the hurdle for effective immune control of cells reactivated from latency may be high ^8, 9^.

Why cells carrying HIV proviruses continue to expand during ART without expressing viral proteins in the process remains an unresolved paradox. To solve this conundrum tremendous effort is going into characterizing the latent virus reservoir and understanding ongoing immune activation during ART. It is hoped that a synthesis of findings in both areas may provide important clues about navigating available and newly arising treatment options toward a cure.

## Mucosal effects of tenofovir

A third area that has been little considered is the effect of ART drugs, both on viral latency and immune activation. Modern antiretroviral combination therapy provides tremendous clinical benefits for HIV-infected patients, dramatically improving quality of life and prolonging life expectancy. Thus, the possibility that a component of ART could paradoxically decrease the chance of a cure being effective had never crossed my mind when we initiated a systems biology evaluation of an ART drug topically applied to the rectal mucosa in a phase I clinical safety trial. This trial, MTN-007 ( www.clinicaltrials.gov/ct2/show/NCT01232803), tested the safety and tolerability of a gel containing 1% tenofovir, a phosphonated nucleoside reverse transcriptase inhibitor (NRTI) in development for potential use to prevent rectal HIV transmission ^10^. A gel containing 2% nonoxynol-9 (N-9) (a temporary mucosal toxin) was included as a positive control arm, and hydroxyethyl cellulose gel and no gel served as negative controls. Our original hypothesis was that the effects of 1% tenofovir gel on the mucosal transcriptome would be negligible whereas N-9 would activate inflammatory genes. However, upon unblinding of the microarray data, we were surprised to find that tenofovir caused many more genes to change than N-9, more often suppressing than enhancing gene expression ^11^.

Tenofovir caused three particular changes that bear potential relevance to the HIV cure agenda. First, it strongly inhibited the transcription of a large number of nuclear transcription factors; second, it inhibited the anti-inflammatory function of mucosal epithelial cells; and third, it stimulated signatures of increased cell proliferation and viability. Results obtained from rectal biopsies were replicated in primary vaginal epithelial cells, which also proliferated significantly faster in tenofovir’s presence. In addition to the breadth of transcriptional changes, individual effects caused by tenofovir could be large. For example, both *in vivo* and *in vitro*, the drug blocked transcription and protein production of interleukin 10 (IL-10) in the range of 90% ^11^.

## An emerging hypothesis

**Figure 1.**
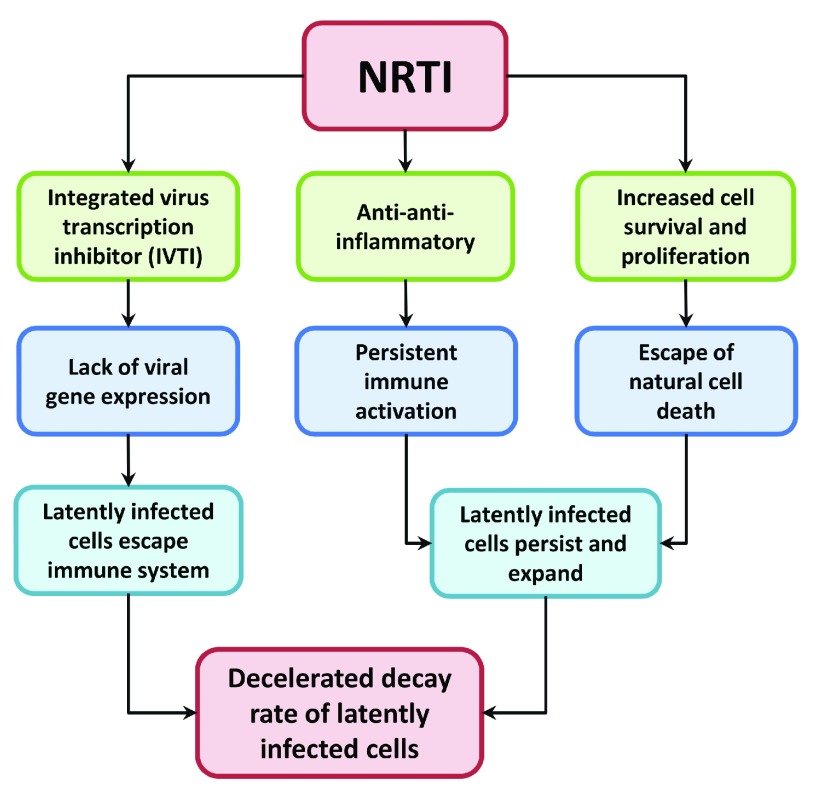
Hypothesized effects of nucleoside reverse transcriptase inhibitors (NRTI) on HIV latency.

From these data grew my first suspicion that tenofovir, and perhaps more generally NRTIs, could have unappreciated effects on HIV latency, and may in fact prevent HIV cure by promoting the survival of cells with integrated provirus ( Figure 1). Before developing this concept below, I want to caution that many of the statements are preliminary and/or hypothetical, intended to serve as a stimulus to the field for further investigation and verification.

Based on the pronounced inhibitory activity of tenofovir on the transcription of many genes, I hypothesize that it also inhibits transcription of provirus integrated into such genes. Host gene transcriptional activity has been shown to be an important determinant of integrated HIV transcription ^12^. This ‘integrated virus transcription inhibitor’ (IVTI) effect of tenofovir and other NRTIs could explain the transcriptional silence of integrated provirus during ART, since nearly all patients receive an ART regimen containing not just one but two NRTI drugs. Tenofovir’s IVTI activity is supported by the preliminary finding that genes reported in two recent studies to be preferential sites of HIV integration after periods of ART appear to overlap with genes inhibited in our studies by tenofovir ^3, 4^. A preliminary analysis of the lists of genes highlighted in the two papers and those found to be strongly inhibited by tenofovir in the rectum showed considerable overlap, including CREBBP, IL6ST, KIF1B, FBXW7, DDX6, IKZF3, ZNF652, DST, CLIC5, GRB2, CEPT1, TAOK1 and PAK2. No overlap was found with genes inhibited by N-9. If true, this overlap would imply that over time NRTIs select for cells in which latent HIV survives because of the drugs’ inhibitory effects on transcription of genes hosting integrated provirus.

The IVTI function of NRTIs could be complemented in favoring latency by their inhibitory effect on the immune system’s anti-inflammatory circuits. In our study, tenofovir was not directly inflammatory, but its strong inhibition of IL-10, as well as of pathways downstream of the immune homeostatic factor TGF-β, indicated that once inflammation is triggered by an outside event, which could be HIV infection itself, it could be prolonged or perpetuated in the presence of tenofovir. I call this the ‘anti-anti-inflammatory’ action of tenofovir.

In our cohort of individuals at low risk for inflammation and HIV infection, we did not detect overt inflammation, although tenofovir did significantly increase the density of CD3 ^+^ and CD7 ^+^ lymphocytes in the rectal mucosa. The participants in CAPRISA 004 ( www.clinicaltrials.gov/ct2/show/NCT00441298), an efficacy trial that demonstrated an overall 39% protective effect of vaginal 1% tenofovir gel ^13^, were at much higher risk for inflammation and HIV infection. This may have uncovered an interesting paradoxical effect of tenofovir: unpublished data in a subset of CAPRISA 004 participants suggest that in the presence of inflammation the risk of HIV infection increased markedly more in the tenofovir than the placebo arm (personal communication). Further analyses by CAPRISA 004 investigators are ongoing. If confirmed, I would hypothetically attribute this effect to tenofovir’s anti-anti-inflammatory action.

Thus, the anti-anti-inflammatory effect of NRTIs could explain why the massive immune activation caused by primary HIV infection never completely reverses despite effective ART. Interestingly, a similar persistence of immune activation is observed in HSV infection treated with acyclovir, a nucleoside analogue related to NRTIs, which also inhibits DNA synthesis by terminating the growing strand. That HSV-induced local immune activation does not resolve well with acyclovir treatment has been identified as a possible reason why the HSV-associated increase in HIV susceptibility does not reverse when women with genital HSV infection receive acyclovir ^14– 16^. Perhaps acyclovir has some of the same anti-anti-inflammatory properties as tenofovir.

Residual immune activation perpetuated by NRTIs could drive the ongoing expansion of cells harboring integrated provirus, and their IVTI function could simultaneously limit transcription of these proviruses. Indeed, our analysis so far indicates that the genes generally turned on by cell activation and the HIV-hosting genes inhibited by tenofovir are different, potentially explaining this apparent paradox. Additionally, the direct cell proliferation- and viability-enhancing effects of tenofovir could contribute to the persistence and expansion of latently infected cells.

## ART and anatomic sites of HIV latency

In theory, the HIV latency-inducing effects of NRTIs would likely be strongest where drug concentrations are highest *in vivo*. Studies on tenofovir’s biodistribution after oral administration show that it highly enriches in gut tissues ^17– 19^, which is believed to harbor a major portion of the latent HIV reservoir ^20^. Estimates also indicate that the rectal concentrations of tenofovir diphosphate, the active intracellular metabolite, are comparable after seven days of oral tenofovir dosing or a single dose of intrarectal 1% tenofovir gel (personal communication, Dr. Craig Hendrix, Johns Hopkins University). Thus, it is likely that some of the effects we observed in the rectal mucosa after topical application also occur after oral dosing, in particular with years of administration and in combination with a second NRTI.

Of note, after oral dosing, NRTI drug concentrations may be even higher in the small intestine than in the colon and rectum, because in the upper gastrointestinal tract locally dissolving drug likely adds to drug distributing from the blood stream. If NRTIs do indeed promote latency, then high drug concentrations would make the small intestine favorable for HIV latency, consistent with the observation that within the gut the duodenum and ileum were preferential sites of residual HIV DNA and unspliced RNA in ART-suppressed patients ^20, 21^. In fact, if NRTIs did not enhance latency, it would be difficult to explain why residual HIV is found precisely where antiretroviral drug concentrations are highest.

## Two special cases of cure without ART

Circumstantial evidence suggests that pharmacological ART is not required to cure HIV/simian immunodeficiency virus (SIV) infection. The only adult patient ever cured of HIV infection, the “Berlin patient” Timothy Brown, received a stem cell transplant from a donor homozygous for a 32-bp deletion in the CCR5 allele, which provides resistance against HIV-1 infection ^22^. He took suppressive ART until the point of his first stem cell transplant, at which point he stopped all ART and never resumed it. Of course, he received a powerful alternative to pharmacological ART in the form of two CCR5-deficient stem cell transplants, carried out about one year apart. However, he did not achieve complete chimerism for some time after transplantation, because CCR5-expressing macrophages were still present in rectal biopsies 5.5 months following the stem cell transplants ^22, 23^, and thus potential HIV target cells were not completely eliminated at that point. This could have provided a hold for residual HIV. Perhaps removing the hypothetical latency-favoring activity of the NRTI drugs could have contributed to his cure.

In contrast, two HIV-1-infected patients in Boston who also received stem cell transplants continued ART in the peri- and post-transplantation period, and were not cured ^24^. Notably, though, these two patients did not receive CCR5-negative stem cells, which provided a less favorable scenario than in the Berlin patient’s case.

The only animals ever cured from a highly pathogenic SIV infection were rhesus macaques who had been vaccinated before SIV challenge with SIV-protein-expressing rhesus cytomegalovirus vectors ^25^. Although the vaccinated rhesus macaques all showed signs of ongoing systemic infection for weeks or months after challenge, protected monkeys lost all indications of SIV infection over time, consistent with immune-mediated clearance of an established lentivirus infection. None of these animals ever received ART.

While it was suggested that establishment of a latent SIV reservoir might have been prevented by the persistently high frequencies of vaccine-induced SIV-specific CD8 ^+^ T lymphocytes, early on many of these animals showed clear signs of productive infection, which requires viral integration. Thus, a latent reservoir was likely established. However, in the absence of the latency-prolonging effects of NRTIs the decay rate of provirus-containing cells could hypothetically have been accelerated, due to faster natural cell death, less cell expansion, and higher expression of viral proteins, allowing immune recognition by the SIV-specific cytolytic T cells. No viral blips were detected in any animals beyond 70 weeks, perhaps offering a clue as to the time frame required to eradicate a latent reservoir in the absence of NRTIs. However, the pool of latently infected cells was likely small in these animals, and eradication of a larger reservoir may take longer.

## Conclusion and outlook

In summary, given that (1) NRTIs may prevent immune detection of latently infected cells by inhibiting transcription of integrated virus, (2) NRTIs may increase persistence of cells with integrated virus by perpetuating inflammation and enhancing cell proliferation, (3) the only monkeys ever cured of SIV infection never received ART, and (4) the only adult patient ever cured of HIV infection discontinued ART before initiating another powerful antiviral therapy, I hypothesize that effectively suppressing HIV with a strategy that does not contain an NRTI component has curative potential.

Only a few years ago, finding a similarly suppressive alternative to an NRTI-containing ART regimen would have posed a dilemma ^26^. Today, powerful second-generation integrase inhibitors and non-NRTI drugs (NNRTIs) are entering early human clinical trials ^27– 29^. Active vaccination, passively infused neutralizing antibodies and vector-expressed CD4/CCR5 co-mimetics show promise as therapeutic immune interventions ^25, 30– 34^, and even more complex strategies such as HIV receptor deletion and specific destruction of integrated viral DNA sequences are progressing ^35^. We are thus moving into a phase where effective NRTI-sparing strategies are becoming reality and could offer hope for a cure.

One phase IIb trial ( www.clinicaltrials.gov/ct2/show/NCT02120352) is registered to switch HIV-1-infected patients who are initially suppressed with an NRTI-containing regimen to an NRTI-free combination of GSK744 LA, a long-acting injectable formulation of the novel integrase inhibitor GSK1265744 ^27^, and TMC278 LA, a long-acting injectable formulation of the novel NNRTI TMC278 (ripilvirine) ^29^. Though not designed to test a cure, this regimen may in fact have curative potential. The study sponsors should consider adjusting their design for that purpose.
