# Supplementary material for: A new hypothesis on HIV cure
Source: F1000Res. 2015 Mar 24;4:77. [Version 1] doi: 10.12688/f1000research.4529.1 (PMC4560250; doi:10.12688/f1000research.4529.1)
Supplement: Peer review history

# Peer review: Referee response for version 1

**DOI:** 10.5256/f1000research.4843.r8703

**Published:** 2015-08-24

**Authors:** Leonid Margolis

**Affiliations:** 1 National Institute of Child Health and Human Development, National Institutes of Health, Bethesda, MD, USA

This work by Hladik addresses one of the most important problems of HIV research: the persistence of a “latent reservoir” in spite of the virtually complete suppression of viral replication. Indeed, despite dramatic progress in the “functional” cure of HIV infection, we have failed to eradicate virus from the infected organism. The author suggests that the nucleoside reverse transcriptase inhibitors (NRTIs), in particular tenofovir, have a side effect: they suppress a natural anti-inflammatory activity, thus, probably indirectly, facilitating inflammation. Also, ART does not prevent cell proliferation and in some cases even facilitates it, thus increasing the number of cells with the integrated provirus. This proliferation may contribute essentially to the establishment and increase of the HIV reservoir.

The hypothesis suggested in this paper was not tested directly, but the author presents significant arguments in favor of it.  His arguments are based on his and others’ research on the effect of tenofovir on tissue explants. One of the strongest effects of tenofovir on human tissues is a  blockade of transcription and protein production of IL-10.  Moreover, analysis of gene activation in rectal tissue of patients under ART led the author to imply that because of the drugs’ inhibitory effects on transcription of genes hosting integrated provirus, NRTIs select over time for cells in which latent HIV survives.

Finally the author discusses the case of a Berlin patient as well as the curing of non-human primates (rhesus macaques) who have been vaccinated before SIV challenge with SIV protein-expressing rhesus cytomegalovirus vectors.  In none of these unique  examples of cures was ART used.

In summary, the author suggests that ART suppresses anti-inflammatory responses, indirectly promoting inflammation. This may be true not only for tenofovir and HIV but also for acyclovir and HSV, as these drugs, despite suppressing HSV facilitate HIV in the treated individuals.

This is an original and interesting hypothesis that may explain some aspects of HIV infection that are not understood yet. By the way, it may explain why HIV-1 patients under ART never return to normal even if their virus, which has been suppressed for years, is undetectable, while the immune activation persists, probably leading to various AIDS-unrelated diseases and to  premature aging.  The author may further develop his hypothesis to cover the area of age-related diseases in functionally cured patients under ART

On the other hand, it is important for the author to emphasize more strongly the success of modern ART as a universal treatment. Even if the author’s hypothesis be proved, the effects he described would be at most the side-effects of the successful treatment, although very important ones.  Moreover, in my opinion, the establishment and persistence of the HIV reservoir hardly can be explained by only one factor.

Finally, the association of the cure with the lack of ART application in just three cases is of course anecdotal, and this fact should be emphasized more strongly.

All these critical remarks are rather of an editorial nature, and Hladik’s original hypothesis certainly deserves to be indexed and to be tested in targeted basic and epidemiological research.

I have read this submission. I believe that I have an appropriate level of expertise to confirm that it is of an acceptable scientific standard.

# Peer review: Referee response for version 1

**DOI:** 10.5256/f1000research.4843.r8673

**Published:** 2015-06-22

**Authors:** Eric Hunter

**Affiliations:** 1 Department of Pathology and Laboratory Medicine, Emory University School of Medicine, Atlanta, GA, USA

In the absence of a highly effective preventative vaccine to protect against HIV infection, and with a growing burden of HIV-1 infected individuals currently on or soon to be eligible for antiretroviral treatment, there is a major research effort underway to seek approaches that might yield a functional cure for the disease. As Florian Hladik points out in this Opinion Article, a major challenge and obstacle to the goal of developing an HIV Cure is the fact that, despite years of suppressive antiretroviral therapy, a stable latent virus reservoir persists that can seed the rapid rebound in HIV-1 replication. An event that is consistently observed whenever treatment of chronically infected individuals is interrupted.

In this Opinion Article, Hladik draws on recent research from his laboratory, in which a systems biology approach was taken to understand the impact of a topically applied nucleoside reverse transcriptase inhibitor (NRTI), tenofovir, on gene expression in the rectal mucosa. The results of this study showed that topical tenofovir inhibited the transcription of a large number of nuclear transcription factors, inhibited anti-inflammatory functions of mucosal epithelial cells, in particular IL-10 expression, and stimulated signatures of increased cell proliferation and enhance viability.  Similar results were observed in primary vaginal epithelial cells in culture (author reference 11).

Based on this research, Hladik hypothesizes that tenofovir might paradoxically enhance formation and maintenance of the latent reservoir by inhibiting the transcription of proviruses integrated into the suppressed genes, and that such transcriptional silencing could explain the lack of HIV expression in such cells. Support for this theory comes from the fact that genes shown recently to be preferential sites for HIV-1 integration (refs) overlap with those inhibited by tenofovir.  Moreover, Hladik proposes that by inhibiting anti-inflammatory functions, tenofovir and other NRTIs could perpetuate residual immune activation and drive ongoing expansion of cells harboring an integrated provirus. An effect that could be exacerbated by the cell proliferation and enhancement of viability also observed following administration of the drug.  Because NRTIs are generally delivered orally, the author argues that the above effects would be expected to be strongest where drug concentrations are highest, consistent with observations that residual viral DNA is preferentially found in the duodenum and ileum.

Given the strong base to this interesting hypothesis, it is unfortunate that the author uses somewhat anecdotal examples of situations, where non-NRTI approaches have resulted in apparent “cure” of the latent reservoir, to support his theory.  While the mechanism of latent virus clearance has not been defined, in the first of these, the Berlin patient Timothy Brown, did stop ART, but as pointed out by the author, underwent two CCR5-deficient stem-cell transplants that could through a combination of resistant cells and graft versus host reaction have cleared residual latent cells. The second example of apparent cure from ongoing early infection is that observed with cytomegalovirus SIV vaccine vectors. While approximately 50% of infected vaccinated animals do appear to clear SIV infection, the mechanistic basis for this is unknown, and it would seem to be a stretch to argue that this is simply because the animals were not ART treated.

The hypothesis put forward in this Opinion Article – that drugs highly effective in suppressing viral replication might actually play a role in sustaining, in a latent state, the very virus that it so effectively inhibits – seems at first counter-intuitive.  Nevertheless, the gene expression data from the phase I clinical trial of tenofovir as a rectal microbicide, do provide a plausible underpinning for the hypothesis and it is one that should be tested experimentally.  Indeed, given the diversity of treatment regimens that are ongoing for patients, it seems likely that existing clinical samples may well be available to allow such a study to be performed.  Moreover, as noted by the author, clinical trials that have or propose to switch patients from an NRTI-based ART regimen to one lacking NRTIs would provide clinical samples with which to test the hypothesis.

Although not discussed by the authors in the eLife manuscript, if the effect of tenofovir on gene expression is reversible – something that could be tested *in vitro* – then one might anticipate that individuals switching from a NRTI-based regimen to a combination of NNRTI, protease inhibitor, or integrase inhibitor, might be expected to exhibit a more rapid decline in the latent reservoir compared to those remaining on an NRTI regimen.  Moreover, it should be possible using RT-SHIVs to test whether non-NRTI suppressive antiretroviral regimens result in a reduced latent virus reservoir compared to those on a tenofovir-based regimen.

A minor point – in the Abstract the author states “(NRTIs) may prevent human immunodeficiency virus (HIV) cure”. This should be softened to “may reduce the likelihood of a cure for human immunodeficiency virus”, since one cannot predict the efficacy of future “cure” approaches.

Finally, while the hypothesis is novel and if proven may provide clues to limiting the latent viral reservoir in patients on ART, it is important to note that tenofovir and a second NRTI, Emtricitabine (FTC) form the base of the 3 drug regimen that is the low-cost mainstay of initial HIV-1 treatment across the continent of Africa and other developing countries.  While the long-term goal of a cure for HIV, along with an effective preventative vaccine, will likely be key to controlling the epidemic, it is equally important not to undermine confidence in these highly effective components of current therapy.

I have read this submission. I believe that I have an appropriate level of expertise to confirm that it is of an acceptable scientific standard.
